# Prognostic Value of Pre-Treatment Systemic Inflammatory Markers in Pediatric Unilateral Wilms Tumor

**DOI:** 10.3390/cancers18132179

**Published:** 2026-07-07

**Authors:** Hadeel Halalsheh, Lana Amer, Mohammad Alzoubi, Noor F. Al-Assaf, Adam Diab, Nada Odeh, Iyad Sultan

**Affiliations:** 1 Department of Pediatrics, King Hussein Cancer Center, Amman 11941, Jordan; 2 Department of Pediatrics, The University of Jordan, Amman 11942, Jordan; 3 School of Medicine, The University of Jordan, Amman 11942, Jordan; 4 Artificial Intelligence Office, King Hussein Cancer Center, Amman 11941, Jordan

**Keywords:** Wilms tumor, neutrophil-to-lymphocyte ratio, pan-immune-inflammation value, lymphocyte-to-monocyte ratio, prognostic biomarkers

## Abstract

**Background**: Systemic inflammation has been implicated in prognosis of multiple malignancies, yet evidence regarding its role in Wilms Tumor (WT) remains scarce. We investigated the prognostic significance of pre-treatment inflammatory markers in unilateral WT. **Methods**: We conducted a retrospective analysis of children with unilateral WT treated at our institution between November 2014 and December 2023. Clinical characteristics, treatment, and outcomes were evaluated. Inflammatory markers included a novel pan-immune-inflammation value (PIV), neutrophil-to-lymphocyte ratio (NLR), and lymphocyte-to-monocyte ratio (LMR). Optimal cut-off values for Event-Free Survival (EFS) and Overall survival (OS) were determined using data-derived optimization to maximize survival curve separation (surv_cutpoint). Cox proportional hazards models were used for survival analysis, while ROC curves were used to calculate AUC values and confirm optimal cut-off points. **Results**: We included 91 patients (median age: 3.6 years; 62% female), of whom 34% presented with metastasis. Higher EFS was significantly associated with low PIV (cut-off 288.9), low NLR (cut-off 1.1), and high LMR (cut-off 6.3). Similarly, OS was positively associated with lower PIV (HR 8.56 for high PIV, *p* = 0.038) and lower NLR (HR 4.63 for high NLR, *p* = 0.043). ROC analysis confirmed their discriminative ability; when evaluated at the unified survival cutpoints, LMR yielded an AUC of 0.692 for 5-year mortality (at cut-off 6.3), while NLR yielded an AUC of 0.671 for 5-year events (at cut-off 1.1). **Conclusions**: Pre-treatment inflammatory markers demonstrate significant prognostic value in unilateral WT. Elevated NLR and PIV, along with lower LMR, correlate with poorer survival. These accessible biomarkers provide a valuable, low-cost tool for risk assessment. Our optimal thresholds differed from Western adult cohorts, emphasizing the need for population-specific reference intervals in global pediatric oncology.

## Simple Summary

Wilms tumor is the most common childhood renal cancer. While overall survival rates are excellent, identifying which patients are at a higher risk of relapse or poor outcomes remains a clinical challenge. This study investigated whether routine, inexpensive blood tests taken before treatment—specifically markers that measure the body’s immune and inflammatory response—could predict patient outcomes. By analyzing data from 91 children with unilateral Wilms tumor, we found that certain inflammatory profiles, such as a high neutrophil-to-lymphocyte ratio (NLR) and pan-immune-inflammation value (PIV) along with lower lymphocyte-to-monocyte ratio (LMR), are significant indicators of poorer survival and higher relapse risk. These accessible biomarkers could provide a valuable, low-cost tool to better assess risk and personalize treatment plans for children with Wilms tumor.

## 1. Introduction

Wilms tumor (WT) is the most common pediatric renal tumor across all global regions [1]. In recent decades, significant improvements in survival outcomes for patients with WT have been achieved, leading to overall survival (OS) rates exceeding 90% [2]. A fundamental factor driving these improvements is the refined risk stratification facilitated by collaborative working groups like the International Society of Pediatric Oncology (SIOP) and the Children’s Oncology Group (COG). These distinct protocols utilize a risk-based approach where multiple factors, including stage, pathology, and molecular markers (such as loss of heterozygosity at 1p and 16q), play a critical role in determining the necessity for treatment intensification [3,4,5,6,7,8,9]. However, while post-operative histopathological and molecular profiling represent cornerstone advances in WT risk stratification, their dependence on surgical tissue limits their applicability at the point of initial clinical presentation, particularly in low- and middle-income countries where access to molecular diagnostics remains constrained. Consequently, approximately 10–15% of patients still experience relapse, and the prognosis for recurrent WT remains dismal [10]. There is an unmet clinical need for accessible, pre-operative, and non-invasive markers that can identify high-risk patients at the time of initial diagnosis. Identifying such patients early could justify the integration of novel therapeutics or altered surgical approaches prior to definitive resection.

The systemic inflammatory response is well-documented to contribute to cancer initiation, progression, and metastasis. This physiological response affects tumor cell proliferation, alters the tumor microenvironment, and promotes metastasis [11,12]. For example, tumor-associated neutrophils are recognized as powerful promoters of angiogenesis, secreting pro-tumor cytokines that facilitate tumor growth [13]. Accordingly, inflammatory markers and composite scores have emerged as prognostic indicators across numerous solid and hematological malignancies. Scores such as the Glasgow Prognostic Score and the Pan-Immune-Inflammation Value (PIV) have been investigated and shown to serve as independent predictors of survival in adult patients with a range of cancers [14,15,16]. Furthermore, an elevated neutrophil-to-lymphocyte ratio (NLR) and low lymphocyte-to-monocyte ratio (LMR) have been associated with worse outcomes in many adult cancers, including gastric, colorectal, esophageal, breast, and oral cancers [14,17,18,19,20,21,22].

Despite the established role of systemic inflammation in driving tumor progression, the prognostic utility of these inflammatory markers in pediatric tumors overall, and WT specifically, has been largely underexplored. While embryonal pediatric tumors like WT differ biologically from adult carcinomas, the tumor microenvironment’s reliance on immune evasion and angiogenesis remains a universal hallmark of cancer progression [11,12]. Unlike single-lineage markers, novel composite indices like the PIV incorporate the entirety of the peripheral immune response—including neutrophils, platelets, monocytes, and lymphocytes—potentially offering a more comprehensive reflection of tumor-host immune dynamics [17,23,24]. Therefore, this study aims to investigate the prognostic value of pretreatment inflammatory markers, specifically PIV, NLR, and LMR, on survival outcomes in patients with unilateral WT and to establish optimal cut-off values for potential clinical application. A recent study by Cui et al. reported concordant findings in a Chinese pediatric cohort, with PIV, NLR, and stage predicting EFS, and PIV and stage predicting OS [25]. The present study extends this work by additionally evaluating LMR, applying a unified cutpoint strategy across both survival endpoints, and characterizing outcomes in a Middle Eastern Arab pediatric cohort—a population in whom baseline hematological profiles may differ systematically from those previously studied, underscoring the need for region-specific validation of inflammatory biomarker thresholds.

## 2. Materials and Methods

### 2.1. Patient Cohort and Study Design

**Figure 1:**
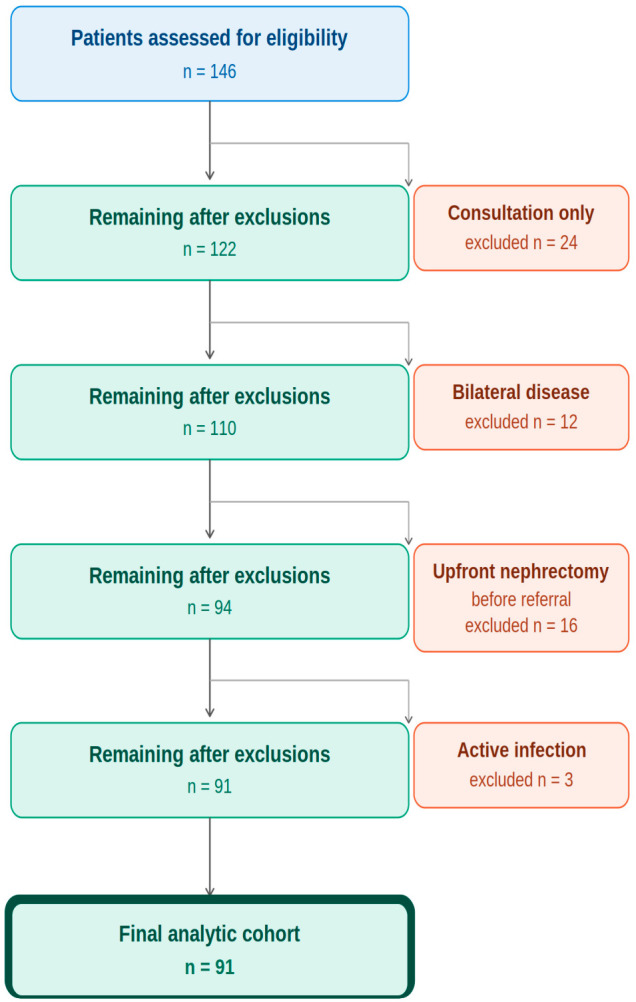
Patient selection flow diagram. Of 146 patients with Wilms tumor assessed for eligibility, 24 were excluded for consultation only, 12 for bilateral disease, 16 for upfront nephrectomy before referral, and 3 for active infection at presentation, yielding a final analytic cohort of 91 patients with unilateral Wilms tumor.

Following King Hussein Cancer Center (KHCC) institutional review board approval (24KHCC196), we performed a comprehensive review of medical records for patients aged ≤ 18 years diagnosed with WT at KHCC, Jordan. Children treated between November 2014 and December 2023 were included in this study. The analysis focused on cases of unilateral disease. Patients with bilateral disease were excluded, as they represent a distinct clinical entity. Furthermore, we excluded individuals referred solely for consultation, radiation therapy, or surgery, as well as those who had undergone nephrectomy or chemotherapy prior to their referral to our institution. To prevent confounding of the baseline inflammatory markers, patients presenting with fever or an active infection at the time of diagnosis were also excluded. The patient selection process, including the number of patients excluded at each stage, is summarized in Figure 1.

All patients were treated according to the International Society of Pediatric Oncology (SIOP) protocol. Laboratory data, including differential white blood cell (WBC) count and platelet count, were obtained at initial presentation as part of the routine baseline workup, prior to the initiation of any neoadjuvant chemotherapy.

### 2.2. Data Collection and Definitions

Demographic and clinical characteristics collected included age at diagnosis, sex, presence of metastasis, tumor stage, histology, and clinical outcomes. Laboratory data obtained at initial presentation, as part of the routine baseline workup prior to any therapeutic intervention, included differential WBC and platelet counts.

Systemic inflammatory markers were calculated using the following established formulas:

- Absolute neutrophil count (ANC) = WBC × neutrophil percentage
- Absolute lymphocyte count (ALC) = WBC × lymphocyte percentage
- Absolute monocyte count (AMC) = WBC × monocyte percentage
- Neutrophil-to-lymphocyte ratio (NLR) = ANC/ALC
- Lymphocyte-to-monocyte ratio (LMR) = ALC/AMC
- Pan-Immune-Inflammation Value (PIV) = (ANC × Platelet count × AMC)/ALC

### 2.3. Statistical Analysis

Demographic, tumor, and treatment characteristics were summarized by descriptive statistics. OS was defined as the time from diagnosis to death from any cause, or to the last follow-up for patients remaining alive. Event-free survival (EFS) was defined as the time from diagnosis to the occurrence of disease recurrence, progression, death, or last follow-up for patients who did not experience an event.

Optimal cut-off values were determined using the surv_cutpoint function (R package survminer, maxstat method), which evaluates the log-rank test statistic at every possible split point of the continuous marker and selects the threshold producing the greatest survival curve separation. This approach is data-driven; however, because cutpoints are derived and tested within the same dataset, performance metrics are subject to optimism bias. A unified set of EFS-optimized cutpoints was applied consistently to both EFS and OS analyses, across all survival analyses, and for reporting sensitivity and specificity in ROC curve evaluations, allowing a single threshold to be used across endpoints. Patients were subsequently stratified into “low” and “high” groups based on these determined thresholds. EFS and OS rates were then compared across these groups and other patient characteristics.

Cox proportional hazards regression was used for univariable and multivariable comparisons of different covariates. The clinical variables age at diagnosis and stage were included as covariates along with inflammatory markers. Variables with *p* ≤ 0.05 in univariable analysis were considered for multivariable models.

Given the mathematical interdependence of PIV, NLR, and LMR—which share ALC in their denominators—pairwise Spearman rank correlations were calculated between all three markers to quantify collinearity prior to survival analysis. In view of the strong inter-correlations identified and the limited number of outcome events (25 EFS events, 16 OS events), the three markers were not entered simultaneously into any single multivariable Cox model. Instead, each inflammatory marker was evaluated independently in univariable analysis. In the multivariable models, inflammatory markers were included individually alongside the pre-specified clinical covariates (age and stage), rather than as a combined set, to avoid variance inflation from collinear predictors.

## 3. Results

### 3.1. Patient Characteristics

The study cohort included 91 patients diagnosed with unilateral WT, presenting with a median age of 3.6 years (range: 0.4–15.1 years); 56 patients (62%) were female. Distribution by stage was as follows: 25 (27%) patients had stage I, 20 (22%) had stage II, 15 (16%) had stage III, and 31 (34%) had stage IV disease. Regarding histology, favorable histology was most common, observed in 64 patients (70.3%), followed by blastemal predominant in 15 (16.5%), and anaplasia in 12 patients—7 (7.7%) with diffuse and 5 (5.5%) with focal anaplasia (Table 1).

### 3.2. Baseline Inflammatory Markers and Correlations

At presentation, the median for WBC was 10.2 × 10^3^/µL and the median platelet count 435.5 × 10^9^/L. The calculated median NLR was 1.6 (IQR: 1.00–3.25), median LMR was 4.2 (IQR: 2.90–6.20), and the calculated median PIV was 630.7 (IQR: 244.5–1242.3). Spearman rank correlations between the three inflammatory markers were strong. PIV and NLR were positively correlated (rho = 0.85, *p* < 0.001). PIV and LMR were inversely correlated (rho = −0.83, *p* < 0.001). NLR and LMR were inversely correlated (rho = −0.84, *p* < 0.001). These strong correlations reflect the shared mathematical structure of the three indices: ALC appears in the denominator of NLR (NLR = ANC/ALC), LMR (LMR = ALC/AMC), and PIV (PIV = ANC × Platelets × AMC/ALC).

### 3.3. Survival Outcomes and Prognostic Value of Biomarkers

The median follow-up period for the whole group was 46.8 months (range, 3.6–121.7 months). The cohort experienced 25 EFS events and 16 OS events. The 5-year EFS rate was 69.0% (± 5.4%), and the 5-year OS rate was 77.2% (± 5.5%).

Univariable analysis of categorical inflammatory markers (using data-derived cutpoints) revealed that higher NLR (>1.1) was strongly associated with poorer EFS (HR 7.92, 95% CI: 1.86–33.74, *p* = 0.005). Similarly, elevated PIV (>288.9) was a significant predictor of worse EFS (HR 5.97, 95% CI: 1.40–25.45, *p* = 0.016), and low LMR (<6.3) correlated with inferior EFS (HR 4.35, 95% CI: 1.02–18.52, *p* = 0.046) (Table 2). Similar associations were observed for OS, with elevated PIV (HR 8.56, 95% CI: 1.12–65.34, *p* = 0.038) and elevated NLR (HR 4.63, 95% CI: 1.05–20.47, *p* = 0.043) correlating with poorer clinical outcomes (Table 3). The derived cutpoints produced imbalanced groups for all three markers: 31 patients (34.1%) had NLR ≤ 1.1; 26 patients (28.6%) had PIV ≤ 288.9; and 23 patients (25.3%) had LMR ≥ 6.3. These distributions—a consequence of the right-skewed nature of all three markers and the behavior of the maxstat algorithm—contribute to the wide confidence intervals observed in the Cox models (e.g., NLR HR 7.92, 95% CI 1.86–33.74) and to the modest specificity in the ROC analysis (27.6–37.9%). It should be noted that the data-derived NLR cutpoint of 1.1 is considerably lower than thresholds reported in adult oncology literature (typically > 2.0–2.5) and lower than the few available pediatric benchmarks. In our cohort, the 25th percentile of the NLR distribution was 1.0, and 60 of 91 patients (65.9%) had NLR > 1.1, meaning two-thirds of the cohort were classified as high risk by this threshold. The biological and clinical interpretation of this low cutpoint is discussed further in Section 4.

In the multivariable Cox regression for EFS using categorical variables, high stage (III-IV) remained the strongest independent predictor of adverse outcomes (HR 4.35, 95% CI: 1.55–12.17, *p* = 0.005). While elevated PIV and NLR maintained high hazard ratios (3.62 and 3.06, respectively), they did not reach independent statistical significance in this model (Table 4). Tumor stage could not be included as a covariate in the multivariable Cox regression for OS (Table 5) due to the complete absence of mortality events among patients with Stage I and II disease. In this limited model, which adjusted only for age, neither age nor the inflammatory markers reached independent statistical significance. However, elevated PIV still demonstrated a notably high hazard ratio (HR 5.75, 95% CI: 0.59–55.94).

**Figure 2:**
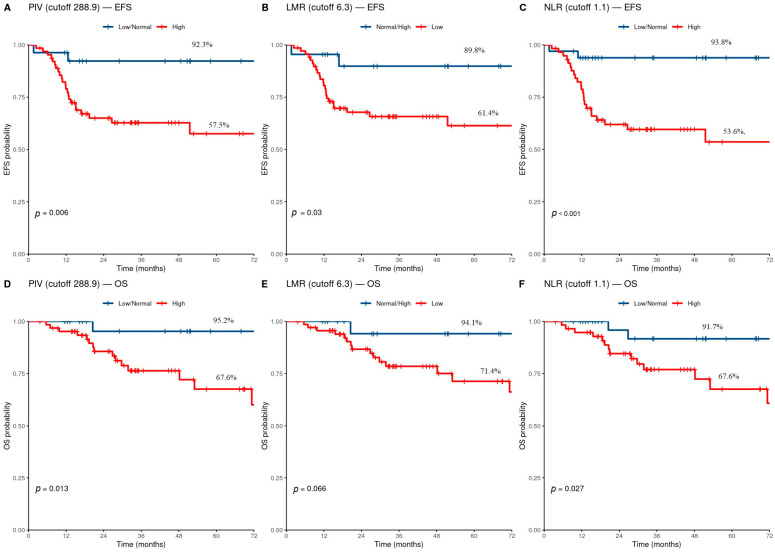
Kaplan–Meier curves for event-free survival (EFS, (**A**–**C**)) and overall survival (OS, (**D**–**F**)), stratified by data-derived cut-off values for pan-immune-inflammation value (PIV, cutoff 288.9; (**A**,**D**)), lymphocyte-to-monocyte ratio (LMR, cutoff 6.3; (**B**,**E**)), and neutrophil-to-lymphocyte ratio (NLR, cutoff 1.1; (**C**,**F**)). Five-year survival estimates and log-rank *p*-values are shown for each comparison.

Kaplan–Meier survival curves, stratified by the calculated optimal cut-off values for each inflammatory marker, revealed significant differences in survival outcomes. For EFS, striking survival differences were seen between the low vs. high PIV groups (5-year EFS: 92.3% vs. 57.5%, log-rank *p* = 0.006; Figure 2A), the high vs. low LMR groups (5-year EFS: 89.8% vs. 61.4%, log-rank *p* = 0.03; Figure 2B), and the low vs. high NLR groups (5-year EFS: 93.8% vs. 53.6%, log-rank *p* < 0.001; Figure 2C). Similarly, significant survival disparities were observed for OS; patients in the low PIV group achieved a 5-year OS of 95.2% compared to only 67.6% in the high PIV group (log-rank *p* = 0.013; Figure 2D). The NLR also demonstrated a significant prognostic split for OS, with the low/normal group reaching a 5-year OS of 91.7% while the high NLR group fell to 67.6% (log-rank *p* = 0.027; Figure 2F). While the LMR followed a similar trend for OS (5-year OS: 94.1% for high LMR vs. 71.4% for low LMR), this specific marker showed a trend toward significance but did not reach the standard threshold (log-rank *p* = 0.066; Figure 2E).

### 3.4. Optimal Cut-Off Values and Predictive Performance

**Figure 3:**
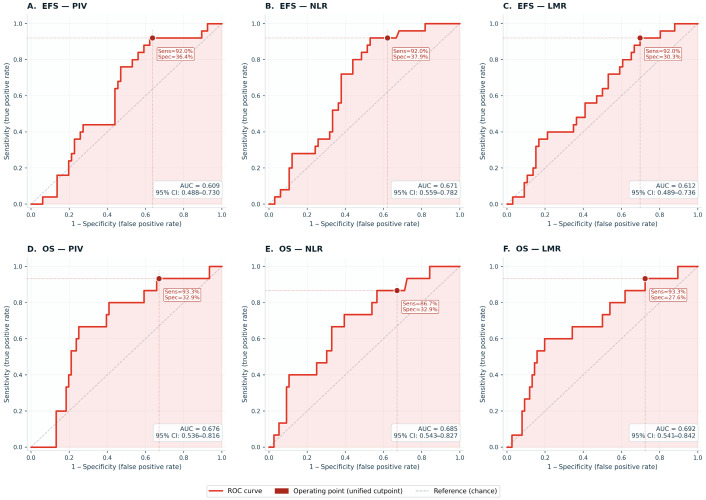
ROC curves for PIV, NLR, and LMR predicting 5-year event-free survival (panels (**A**–**C**)) and 5-year overall survival (panels (**D**–**F**)). AUC with 95% CI is shown in each panel. The filled circle indicates the operating point at the unified survival cutpoint, with corresponding sensitivity and specificity annotated. Dashed diagonal line represents chance-level discrimination.

For predicting 5-year mortality, LMR demonstrated the highest Area Under the Curve (AUC) of 0.692, showing 93.3% sensitivity at the unified cut-off of 6.3. This was closely followed by NLR (AUC 0.685) and PIV (AUC 0.676; 93.3% sensitivity at cut-off 288.9). For the prediction of 5-year disease relapse, NLR yielded the highest AUC of 0.671 with 92.0% sensitivity at a cut-off of 1.1, followed by LMR (AUC 0.612) and PIV (AUC 0.609; 92.0% sensitivity at cut-off 288.9) (Table 6). ROC curves for all three markers for both EFS and OS are presented in Figure 3A–F.

## 4. Discussion

Several prognostic factors have historically been identified in WT, prominently including tumor histopathology, the presence of distant metastases, per-operative tumor volume, and specific molecular alterations [2,26,27,28,29]. While the prognostic utility of inflammatory markers has been extensively validated in adult oncology, there remains a distinct paucity of data regarding their application in pediatric cohorts [14,18,23,24]. This study presents evidence that systemic inflammatory markers, readily assessed via peripheral blood prior to the initiation of any therapy, hold significant prognostic value in children with unilateral WT. These markers not only reflect the baseline inflammatory and immunologic status of the host but also correlate strongly with survival outcomes, functioning as surrogate markers for high-risk disease that can be assessed prior to surgical staging.

Our findings largely corroborate those of Cui et al., who reported that elevated PIV and NLR predicted worse EFS and OS in WT patients treated at a Chinese center [25]. The biological convergence across two geographically and ethnically distinct populations strengthens confidence in the prognostic relevance of these markers. Notable differences exist, however: Cui et al. did not report LMR as a significant predictor, while in our cohort, LMR demonstrated a significant association with EFS on univariable analysis [25]. Differences in institutional treatment protocols, histological distribution, and baseline hematological norms across ethnic groups may account for this discrepancy and deserve further study.

A key methodological consideration in studies evaluating composite inflammatory indices simultaneously is the collinearity inherent to their mathematical structure. In the present cohort, pairwise Spearman correlations between PIV, NLR, and LMR were uniformly strong (rho range: 0.83–0.85), reflecting the fact that ALC appears in the denominator of all three indices. This degree of inter-correlation means that simultaneous entry of all three markers into a single multivariable model would produce unstable and uninterpretable coefficient estimates, particularly given the limited number of outcome events in this cohort. Accordingly, markers were evaluated independently in univariable analysis and not entered as a combined set in multivariable models. The loss of statistical significance for PIV and NLR in the multivariable EFS model—where high stage remained the dominant predictor—most likely reflects two overlapping mechanisms: collinearity between the inflammatory markers themselves, and the established biological relationship between advanced stage and systemic immune perturbation, whereby higher tumor burden drives the neutrophilia, thrombocytosis, and lymphopenia that composite indices capture. This collinearity between stage and inflammatory markers does not diminish the pre-operative clinical utility of PIV and NLR; on the contrary, it confirms that these markers function as accessible surrogates for tumor aggressiveness at a point in time when surgical staging is not yet available.

In the broader oncologic literature, patients presenting with high NLR and PIV and low LMR generally exhibit inferior clinical trajectories. Biologically, these parameters likely represent an underlying immunologic imbalance. Elevated neutrophil or platelet counts, coupled with reduced lymphocyte levels, point toward a tumor-growth-promoting systemic environment. Conversely, relative lymphopenia reflects a profound suppression of the adaptive immune system, specifically a depletion of the cytotoxic CD8+ T-cells necessary for anti-tumor surveillance [30,31].

The identification of the PIV as a powerful risk stratifier is particularly important and represents a highly novel finding in pediatric renal tumors. Combining four parameters—neutrophils, platelets, monocytes, and lymphocytes—PIV offers a comprehensive snapshot of the immune system. Specifically, the incorporation of platelets is crucial; tumor-educated platelets are known to secrete pro-angiogenic factors, shield circulating tumor cells from natural killer (NK) cell-mediated immune destruction, and facilitate extravasation during metastasis. This multi-faceted role in immune evasion and tumor dissemination provides a strong biological rationale for PIV’s superior prognostic depth [32]. Patients in our cohort with an elevated PIV had a markedly reduced 5-year EFS of 57.5%, compared to 92.3% in the low-PIV group. As discussed above, PIV and NLR did not retain independent significance in the multivariable EFS model—a finding best understood through the collinearity framework described earlier—yet their high univariate hazard ratios and exceptional sensitivity (93.3% for 5-year mortality) underscore their value precisely at the moment of initial presentation, before surgical staging is available. The identification of elevated NLR and PIV, alongside low LMR, as specific markers of poor prognosis in our WT cohort aligns with prior observations in other adult solid tumors, suggesting that the fundamental mechanisms of cancer-associated inflammation exceed age and histology [14,17,23].

The clinical utility of our findings is supported by the use of ROC curve analysis and data-derived cutpoints to establish specific, quantifiable cut-off values for both mortality and relapse. It is important to contextualize the predictive performance of these markers. While the ROC analysis demonstrated statistically significant AUC values (ranging from 0.609 to 0.692), these represent moderate discriminative ability. Therefore, pre-treatment inflammatory markers should not be viewed as standalone definitive predictors of relapse or mortality. Rather, they serve as valuable, highly accessible clinical adjuncts intended to complement—not replace—established risk stratification models based on tumor stage, histology, and molecular profiling. However, when comparing our derived thresholds to published data, notable variations emerge. Previous studies exploring inflammatory markers in pediatric solid tumors have frequently reported higher cut-points for NLR, often exceeding 2.0 or 2.5, to predict adverse outcomes. In contrast, our cohort’s data-derived, EFS-optimized cut-point for NLR was relatively lower (1.1). Similarly, optimal LMR thresholds in our analysis differed from established adult and pediatric literature benchmarks. These biomarkers are derived from routine complete blood counts and require no additional cost or specialized infrastructure, making them particularly valuable in low- and middle-income countries where access to molecular diagnostics remains limited.

These discrepancies highlight a critical pathophysiological and epidemiological consideration: while methodological approaches (such as maximum survival curve separation) naturally influence cut-point generation, the baseline hematological and inflammatory profiles of Arab children in the Middle East may inherently differ from those of Western cohorts. Regional environmental factors, endemic subclinical infections, nutritional variations, and distinct immunogenetic backgrounds (such as polymorphisms in cytokine promoter regions) can shift the “normal” inflammatory baseline. Consequently, applying universal, Western-derived cut-points may lead to inaccurate risk stratification in our demographic. This underscores the necessity for population-specific reference intervals rather than assuming a one-size-fits-all threshold for inflammatory biomarkers in global pediatric oncology.

It is important to situate these pre-treatment markers within the full clinical complexity of WT management. Several factors with major prognostic impact are, by definition, unavailable at initial presentation: histological subtype and chemotherapy response are only assessable from the post-nephrectomy specimen; lymph node status depends on intraoperative sampling technique and surgeon decision-making; anaplasia (focal or diffuse) may not be clinically suspected before resection; and chemotherapy can induce downstaging that shortens or alters treatment duration. Pre-treatment inflammatory markers do not replace any of these assessments, nor do they inform them. Their utility lies specifically in the narrow clinical window at initial diagnosis—before imaging results in surgical staging, before histology is available, and before treatment has commenced. In this window, a simple complete blood count may identify patients who are likely to have a turbulent clinical course, warranting heightened clinical vigilance even if formal risk re-stratification must await post-operative information.

To translate these findings into a practical clinical framework, we propose a risk-stratification approach as a hypothesis for prospective evaluation. These markers are particularly relevant in low- and middle-income countries, where access to molecular diagnostics is limited yet a routine complete blood count is universally available at diagnosis, making inflammatory indices a genuinely zero-cost addition to standard workup. At initial presentation, PIV, NLR, and LMR are calculated from the routine pre-treatment complete blood count and integrated with established clinical parameters—tumor stage on imaging, presence of metastasis, and age at diagnosis. Patients with advanced stage (III–IV) and concurrent elevated PIV or NLR may represent a particularly high-risk subgroup in whom closer clinical monitoring during neoadjuvant chemotherapy is warranted, and in whom treating physicians may exercise a lower threshold for treatment modification based on early response assessment. For patients with early-stage disease (I–II), elevated PIV or NLR at diagnosis may identify the minority at unexpected risk for relapse—a group in whom standard staging alone would not prompt heightened vigilance—supporting more frequent post-treatment clinical follow-up and ultrasound-based monitoring beyond the standard schedule. In low- and middle-income settings where resources for uniform intensive follow-up are limited, inflammatory markers at diagnosis could help triage which patients warrant closer surveillance, allowing more efficient allocation of available monitoring capacity. Patients with low PIV, low NLR, and high LMR may conversely represent candidates for treatment de-escalation strategies in future prospective studies, potentially reducing chemotherapy-related morbidity—a consideration of particular importance in resource-constrained settings where long-term toxicity monitoring is less accessible. We emphasize that this framework is hypothesis-generating and requires prospective validation before clinical adoption.

## 5. Study Limitations

Despite the strengths of our analysis, we acknowledge several limitations. The retrospective, single-center design and the relatively modest sample size inherently limit statistical power for detecting highly subtle associations and may affect broad generalizability. The excellent prognosis of early-stage disease at our center resulted in zero mortality events for Stage I and II patients; while this limited the calculation of hazard ratios for stage in the OS model, it underscores the critical need for biomarkers like PIV and NLR to identify the minority of patients at risk for lethal recurrence. Furthermore, a fundamental methodological limitation of this work is the use of data-derived cut-points evaluated within the same dataset from which they were generated. The maxstat method (surv_cutpoint) selects the threshold that maximizes separation of the log-rank statistic across all possible values—an exhaustive search that, by definition, identifies the split most favorable to demonstrating a survival difference in this particular sample. When these same thresholds are then tested in Kaplan–Meier analyses and ROC curves on the same data, the resulting performance estimates (AUC 0.609–0.692; sensitivity 86.7–93.3%) are subject to optimistic bias of unknown magnitude. External validation in an independent prospective cohort—ideally from a different geographic or institutional setting—is essential before these specific thresholds can be recommended for clinical use. We explicitly caution against applying the derived NLR cut-off of 1.1 or PIV cut-off of 288.9 in routine practice without such validation. Our findings are best understood as generating specific, testable hypotheses for future prospective study.

A further limitation is the non-specific nature of these indices. Subclinical infections—including otitis media, urinary tract infections, and low-grade enteritis—can independently produce neutrophilia, monocytosis, and relative lymphopenia, potentially misclassifying non-oncological immune activation as tumor-related risk. Although patients with documented fever or active infection were excluded, subclinical infections without fever were not systematically screened for. Future prospective studies should incorporate baseline CRP or procalcitonin to allow identification and adjustment for occult infection. It should further be noted that these indices are elevated across a broad range of non-malignant inflammatory conditions, including appendicitis and inflammatory bowel disease, confirming that their interpretation as tumor-specific prognostic signals requires careful clinical contextualization and cannot be made in isolation from the full clinical picture.

## 6. Conclusions

Pre-treatment systemic inflammatory markers represent highly valuable, biologically relevant prognostic tools in the management of unilateral WT. Elevated indices of NLR and PIV are significantly associated with worse survival outcomes, whereas higher LMR indicates a more favorable prognosis. Because these biomarkers are readily accessible, universally available, and highly cost-effective, they should be strongly considered for inclusion in future risk-stratification frameworks to guide personalized treatment approaches in pediatric oncology. However, a critical caveat must accompany these findings: the specific cutpoints derived here (NLR > 1.1, PIV > 288.9, LMR < 6.3) were generated and tested within the same dataset, introducing optimistic bias of uncertain magnitude into all performance metrics. These thresholds are strictly hypothesis-generating and must not be applied in routine clinical decision-making until independently validated in prospective, preferably multicenter, cohorts. Practically, children presenting with elevated NLR or PIV at diagnosis may be identified as higher-risk prior to surgical staging, supporting closer post-treatment clinical follow-up and ultrasound-based monitoring—particularly valuable in low- and middle-income settings where targeted surveillance allows more efficient use of available resources.

## Figures and Tables

**Table 1:** Characteristics of patients and tumors.

Variable	Number (Percentage)
Total patients	91
Age at diagnosis median (range)	3.6 years (0.4–15.1)
Sex	
Female	56 (62)
Male	35 (38)
Histologic subtypes	
Favorable histology	64 (70.3)
Blastemal predominant	15 (16.5)
Focal anaplasia	5 (5.5)
Diffuse anaplasia	7 (7.7)
Stage	
Stage I	25 (27)
Stage II	20 (22)
Stage III	15 (16)
Stage IV	31 (34)
NLR median (IQR)	1.6 (1.0–3.25)
LMR median (IQR)	4.2 (2.9–6.2)
PIV median (IQR)	630.7 (244.5–1242.3)
NLR ≤ 1.1/>1.1	31 (34.1)/60 (65.9)
PIV ≤ 288.9/>288.9	26 (28.6)/65 (71.4)
LMR ≥ 6.3/<6.3	23 (25.3)/68 (74.7)

Abbreviations: IQR, interquartile range; PIV, pan-immune-inflammation value; LMR, lymphocyte-to-monocyte ratio; NLR, neutrophil-to-lymphocyte ratio.

**Table 2:** Univariate cox regression—event-free survival.

Variable	Level	HR (95% CI)	*p*-Value
Age at diagnosis	Per year	1.12 (1.01–1.25)	0.040
Overall stage	III/IV vs. I/II	4.78 (1.79–12.75)	0.002
Histology	DA vs. others	2.44 (0.65–9.09)	0.184
Histology	FA vs. others	1.16 (0.22–5.96)	0.863
Histology	FH vs. others	0.61 (0.22–1.71)	0.348
PIV	>288.9 vs. ≤288.9	5.97 (1.40–25.45)	0.016
LMR	<6.3 vs. ≥6.3	4.35 (1.02–18.52)	0.046
NLR	>1.1 vs. ≤1.1	7.92 (1.86–33.74)	0.005

Abbreviations: HR, hazard ratio; CI, confidence interval; DA, diffuse anaplasia; FA, focal anaplasia; FH, favorable histology; PIV, pan-immune-inflammation value; LMR, lymphocyte-to-monocyte ratio; NLR, neutrophil-to-lymphocyte ratio. Age was modeled as a continuous variable; the HR represents the proportional increase in hazard for each additional year of age at diagnosis. Sensitivity analysis dichotomising age at 4 years confirmed the prognostic direction (EFS: HR 2.99, 95% CI 1.32–6.78, *p* = 0.009).

**Table 3:** Univariate cox regression—overall survival.

Variable	Level	HR (95% CI)	*p*-Value
Age at diagnosis	Per year	1.17 (1.03–1.35)	0.020
Histology	DA vs. others	3.48 (0.87–13.99)	0.079
Histology	FA vs. others	0.66 (0.07–5.96)	0.714
Histology	FH vs. others	0.32 (0.09–1.12)	0.074
PIV	>288.9 vs. ≤288.9	8.56 (1.12–65.34)	0.038
LMR	< 6.3 vs. ≥6.3	5.42 (0.72–41.13)	0.102
NLR	>1.1 vs. ≤1.1	4.63 (1.05–20.47)	0.043

Abbreviations: HR, hazard ratio; CI, confidence interval; DA, diffuse anaplasia; FA, focal anaplasia; FH, favorable histology; PIV, pan-immune-inflammation value; LMR, lymphocyte-to-monocyte ratio; NLR, neutrophil-to-lymphocyte ratio. Note: hazard ratios for stage could not be computed for overall survival due to zero deaths occurring in Stage I/II patients. Age was modeled as a continuous variable; the HR represents the proportional increase in hazard for each additional year of age at diagnosis. Sensitivity analysis dichotomising age at 4 years confirmed the prognostic direction (OS: HR 2.71, 95% CI 0.98–7.48, *p* = 0.055).

**Table 4:** Multivariate cox regression—event-free survival.

Variable	Level	HR (95% CI)	*p*-Value
Age at diagnosis	Per year	1.04 (0.91–1.18)	0.552
Stage	III/IV vs. I/II	4.35 (1.55–12.17)	0.005
PIV	>288.9 vs. ≤288.9	3.62 (0.47–27.68)	0.216
LMR	<6.3 vs. ≥6.3	0.70 (0.10–5.02)	0.723
NLR	>1.1 vs. ≤1.1	3.06 (0.51–18.40)	0.222

Abbreviations: HR, hazard ratio; CI, confidence interval; PIV, pan-immune-inflammation value; LMR, lymphocyte-to-monocyte ratio; NLR, neutrophil-to-lymphocyte ratio.

**Table 5:** Multivariate cox regression—overall survival.

Variable	Level	HR (95% CI)	*p*-Value
Age at diagnosis	Per year	1.12 (0.97–1.29)	0.136
PIV	>288.9 vs. ≤288.9	5.75 (0.59–55.94)	0.132
NLR	>1.1 vs. ≤1.1	1.53 (0.27–8.65)	0.630

Abbreviations: HR, hazard ratio; CI, confidence interval; PIV, pan-immune-inflammation value; NLR, neutrophil-to-lymphocyte ratio.

**Table 6:** ROC analysis of 5-year binary outcomes (PIV, LMR, NLR). Sensitivity and specificity evaluated at the unified survival cutpoint value.

Outcome	Marker	AUC (95% CI)	Sensitivity (%)	Specificity (%)
5-year death	LMR	0.692 (0.541–0.842)	93.3	27.6
5-year death	NLR	0.685 (0.543–0.827)	86.7	32.9
5-year death	PIV	0.676 (0.536–0.816)	93.3	32.9
5-year event	NLR	0.671 (0.559–0.782)	92.0	37.9
5-year event	LMR	0.612 (0.489–0.736)	92.0	30.3
5-year event	PIV	0.609 (0.488–0.730)	92.0	36.4

Abbreviations: PIV, pan-immune-inflammation value; LMR, lymphocyte-to-monocyte ratio; NLR, neutrophil-to-lymphocyte ratio.

## Data Availability

The data presented in this study are available on reasonable request from the corresponding author.

## Abbreviations

ALC	Absolute Lymphocyte Count
AMC	Absolute Monocyte Count
ANC	Absolute Neutrophil Count
AUC	Area Under the Curve
COG	Children’s Oncology Group
EFS	Event-Free Survival
KHCC	King Hussein Cancer Center
LMR	lymphocyte-to-monocyte ratio
NLR	neutrophil-to-lymphocyte ratio
OS	Overall survival
PIV	Pan-Immune-Inflammation Value
ROC	Receiver Operating Characteristic
SIOP	International Society of Pediatric Oncology
WBC	White Blood Cell Count
WT	Wilms Tumor
